# Hemorrhage from the Superior Maxilla

**Published:** 1858-07

**Authors:** Isaiah Forbes

**Affiliations:** St. Louis.


### SELECTED ARTICLES.

### ARTICLE X.

*Hemorrhage from the Superior Maxilla.* Reported by Dr. Isaiah Forbes, St. Louis.

On my return from the Dental Convention, I was called to visit the daughter of Mr. W. C. E., who had ruptured a blood vessel in the superior maxilla.

*Appearance of the Mouth and Child.*—The four permanent incisors and first molar were grown to their natural size; the deciduous cuspid and stomach teeth still remained in their natural position. A sack was formed, involving the whole palatine arch, distending down below the line of the teeth, filled with fetid blood; the child was very weak, and the face extremely pallid from previous loss of blood; an incision had been made in the most pendant portion of the sack, for the purpose of discharging the blood it contained. (In my opinion, a very injudicious course of treatment, for the mucous membrane should always be left unbroken, as nature formed it, as the treatment in this case will prove, and which I have invariably adopted in my practice.)

*Cause of the Rupture.*—The child was playing on the veranda (to which the outer railing had not yet been attached,) and backed off, falling some sixteen feet, striking her chin against the stone wall, which surrounds the area of the basement, in consequence of which, the concussion of the temporary teeth was so great against the permanent bicuspids (the roots of which being but partially formed) that the end of a fang ruptured a blood vessel supplying those teeth; my opinion that such was the case, was predicated on the fact, that whenever she was indulged in any food, requiring some degree of pressure to masticate, hemorrhage again ensued.

*Treatment.*—I extracted the anterior deciduous molars, and, with my pointed bistoury, directed by the inner grinding edge of the bicuspid, I made an incision into the sack (which would, of course, be the most dependent point when the parts were pressed to their normal condition,) which I thoroughly cleansed with tepid water, and then injected a weak solution of chloride of zinc, and pressed the distended membrane to its proper position, retaining it there by a compress, formed out of a cork to fit the palatine arch, and covered with muslin, which was kept saturated by diluted tincture of myrrh. The compress extended a little below the line of the teeth, and to avoid bandaging the head, it was secured by a small piece of wood extending across the teeth, which the little patient soon learned to retain in its place, and even to sleep with it in her mouth, with very little effort or inconvenience, taking it out only at meal times.

The patient was of a scrofulous habit, consequently she could not be put on simple diet, but was indulged with the richest food and plenty of it, made up in the form of jellies, &c., and by strict attention was restored to her wonted health and spirits.—*Am. Dent. Review.*

